# Deletion of *Fmr1* results in sex‐specific changes in behavior

**DOI:** 10.1002/brb3.800

**Published:** 2017-08-25

**Authors:** Suzanne O. Nolan, Conner D. Reynolds, Gregory D. Smith, Andrew J. Holley, Brianna Escobar, Matthew A. Chandler, Megan Volquardsen, Taylor Jefferson, Ashvini Pandian, Tileena Smith, Jessica Huebschman, Joaquin N. Lugo

**Affiliations:** ^1^ Department of Psychology and Neuroscience Baylor University Waco TX USA; ^2^ Texas College of Osteopathic Medicine University of North Texas Health Science Center Fort Worth TX USA; ^3^ Institute for Biomedical Studies Baylor University Waco TX USA

**Keywords:** anxiety, autism, Fragile X syndrome, phenotypes, plasticity

## Abstract

**Objective:**

In this study, we used a systemic *Fmr1* knockout in order to investigate both genotype‐ and sex‐specific differences across multiple measures of sociability, repetitive behaviors, activity levels, anxiety, and fear‐related learning and memory.

**Background:**

Fragile X syndrome is the most common monogenic cause of intellectual disability and autism. Few studies to date have examined sex differences in a mouse model of Fragile X syndrome, though clinical data support the idea of differences in both overall prevalence and phenotype between the sexes.

**Methods:**

Using wild‐type and systemic homozygous *Fmr1* knockout mice, we assessed a variety of behavioral paradigms in adult animals, including the open field test, elevated plus maze, nose‐poke assay, accelerating rotarod, social partition task, three‐chambered social task, and two different fear conditioning paradigms. Tests were ordered such that the most invasive tests were performed last in the sequence, and testing paradigms for similar behaviors were performed in separate cohorts to minimize testing effects.

**Results:**

Our results indicate several sex‐specific changes in *Fmr1* knockout mice, including male‐specific increases in activity levels, and female‐specific increases in repetitive behaviors on both the nose‐poke assay and motor coordination on the accelerating rotarod task. The results also indicated that *Fmr1* deletion results in deficits in fear learning and memory across both sexes, and no changes in social behavior across two tasks.

**Conclusion:**

These findings highlight the importance of including female subjects in preclinical studies, as simply studying the impact of genetic mutations in males does not yield a complete picture of the phenotype. Further research should explore these marked phenotypic differences among the sexes. Moreover, given that treatment strategies are typically equivalent between the sexes, the results highlight a potential need for sex‐specific therapeutics.

## 1 INTRODUCTION

Fragile X syndrome (FXS) is a neurodevelopmental disorder caused by a trinucleotide (CGG) repeat expansion in the *FMR1* gene coding for fragile x mental retardation protein (FMRP). The trinucleotide repeats result in hypermethylation of the promoter, which functionally silences *FMR1* and eliminates FMRP synthesis. FXS is phenotypically characterized by intellectual disability, but may extend to include a broad spectrum of behavioral disturbances, including hyperactivity and fidgeting behaviors (Hagerman, Jackson, Levitas, Rimland, & Braden, 1986). FXS is highly comorbid with other neurodevelopmental disorders, such as attention deficit hyperactivity disorder (Wheeler et al., 2014), epilepsy (Berry‐Kravis, 2002), and autism spectrum disorder (ASD) (Clifford et al., 2007). Epidemiological data suggest that *FMR1* mutations have a prevalence rate of 2–6% in the ASD population (Kaufmann et al., 2004).

Sex plays a significant role in the overall prevalence and clinical presentation of FXS. Prevalence rates vary by sex in FXS, with lifetime incidence of approximately 1:4,000 males, as compared to 1:8,000 females (Pembrey, Barnicoat, Carmichael, Bobrow, & Turner, 2001). In terms of characteristics of a behavioral phenotype, sex is also a significant contributor to the clinical presentation of FXS, with male individuals showing more severe behavioral impairment as compared to females. Given that X‐linked disorders often follow a sex‐dependent pattern of symptom severity, this difference has been generally attributed to compensation by the second unaffected X chromosome in females (Germain, 2006; Kazdoba, Leach, Silverman, & Crawley, 2014). However, it has recently been hypothesized that the symptomatology of affected females may be qualitatively different than affected males. Males display higher rates of ASD‐like behaviors (Reiss & Freund, 1992), hyperactivity, and inattentiveness (Hagerman & Sobesky, 1989). In contrast, affected females carry a higher risk for schizophrenia and extreme shyness, but lower risk for intellectual impairment (Reiss, Hagerman, Vinogradov, Abrams, & King, 1988). Deficits in affective processes are also more prevalent among FXS females (Hagerman & Sobesky, 1989).

Despite these established differences in both prevalence and phenotypic severity in humans, the majority of *Fmr1* knockout (KO) studies focus exclusively on males, leaving the influence in females less understood. There is some evidence of a differential phenotype among the sexes, as male *Fmr1* KOs exhibit a reduced anxiety phenotype, whereas females KOs show normal levels of anxiety (Qin, Kang, & Smith, 2005). However, previous studies show mainly similar deficits between male and female *Fmr1* KOs on tests of activity levels, learning and memory (Baker et al., 2010; Ding, Sethna, & Wang, 2014), sensorimotor gating (Baker et al., 2010; Ding et al., 2014), and seizure susceptibility (Nguy & Tejada‐Simon, 2016; Qin et al., 2005) in adulthood. Recent experimental evidence has also shown that female *Fmr1* KOs present normal fear learning and anxiety, but show impaired fear memory (Nguy & Tejada‐Simon, 2016). Furthermore, analysis of behaviors at different ages has shown sex‐specific differences in ultrasonic vocalization production (Reynolds, Nolan, Jefferson, & Lugo, 2016), though other strains have not shown this effect (Gauducheau et al., 2017). In a recently published review, authors summarized the effects of *Fmr1* deletion across both male and female mice, noting that some behaviors have sex‐specific effects, though most behaviors have not yet been examined in females (Romano, Cosentino, Laviola, & De Filippis, 2016).

The effect of homozygous deletion of *Fmr1* in female mice on repetitive behavior, motor coordination, and social behavior remains unexamined. Lack of females in empirical research is especially prevalent in the fields of neuroscience and biomedical studies (Beery & Zucker, 2011), and only in recent years has there been a push to include females. The omission of females broadly across studies seems to stem from the belief that female mammals have a higher degree of intrinsic variability, likely due to estrus cycles. A recent meta‐analysis of 293 articles found that variability was not greater in females for behavioral, morphological, physiological, and molecular traits when they did not account for the estrous cycle when compared with males (Prendergast, Onishi, & Zucker, 2014). Given the omission of female *Fmr1* KO mice from previous phenotypic characterizations and the broad implications of this exclusion, our study aims to further characterize this model by investigating sex‐specific differences by direct comparison of male and female *Fmr1* KOs on tests of activity levels, anxiety behaviors, social behaviors, repetitive behaviors, and motor coordination, as well as hippocampal‐ and amygdala‐based memory.

## 2 METHODS AND MATERIALS

### 2.1 Animals

Male and female *FVB.129P2‐Pde6b+ Tyrc‐ch Fmr1tm1Cgr/*J (Jackson Labs Stock No: 004624) mice were bred to generate wild‐type (WT) and homozygous *Fmr1* knockout (KO) groups for this study. We bred heterozygous dams with wild‐type males to produce homozygous knockout males, wild‐type males, and wild‐type females. We bred heterozygous dams with knockout males to produce homozygous knockout males and females. Offspring toe clippings were preserved in 70% ethanol and sent for genotyping to Mouse Genotype (Escondido, CA, USA). All animals were bred and housed at Baylor University. Following maturation to PD21, animals were weaned into home cages with up to five littermates. The environment was maintained at an ambient temperature, with 12‐hr light and 12‐hr dark diurnal cycles and *ad libitum* access to food and water. All behavioral testing was conducted during the light phase of the cycle, specifically between 9 am and 5 pm. All procedures were performed in accordance with Baylor University Institutional Care and Use Committee and the Guide for the Care and Use of Laboratory Animals of the National Institutes of Health.

All testing was conducted after the mice reached adulthood, approximately 2 months of age, and they were divided into two cohorts (Cohort 1: n_male wildtype_ = 17, n_female wildtype_ = 13, n_male knockout_ = 16, n_female knockout_ = 16; Cohort 2: n_male wildtype_ = 12, n_female wildtype_ = 13, n_male knockout_ = 16, n_female knockout_ = 17) for the purpose of diversifying the sample and minimizing the effects of multiple test exposure. Each cohort received a battery of behavioral testing that was ordered from least invasive to most invasive to minimize test order effects (McIlwain, Merriweather, Yuva‐Paylor, & Paylor, 2001). The first cohort was tested in the following order: open field, elevated plus maze, marble burying, social chamber, and trace fear conditioning. The second cohort was tested in the following order: light–dark box, nose‐poke assay, accelerating rotarod, social partition, and delayed fear conditioning. There was a rest period of 2–3 days between tests. For all testing, the tails of the mice were labeled in order to identify the testing order for the behavioral test. The mice were allowed to acclimate to the testing room for 30 min prior to the beginning of the test.

### 2.2 Activity levels: open field

The open field test was performed to evaluate changes in activity and anxiety levels. The mice were first weighed, then their tails were marked for identification. The open field apparatus consisted of a clear plastic arena (40 × 40 × 30 cm). The lighting and background noise inside the test chamber were kept constant at 100 lux and 60 dB, respectively. During testing, mice were individually placed into the testing arena for 30 min and the experimenter was not present during the testing period. Activity levels were analyzed by a computer‐operated optical animal activity system (Fusion by AccuScan Instruments, Inc., USA). This system also measured other exploratory behaviors such as grooming, rearing, clockwise, and counterclockwise rotations, as well as stereotypic behavior, which accounts for repeated breaking of the same set of beams, for example, during grooming behavior. To evaluate for anxiety behaviors, distance moved and time spent in the center compared to surround region was compared (center was defined as the inner 50% of the field). Following testing, mice were returned to an alternate cage until all mice in their homecage were tested, then all mice were returned to their homecage, and the arena was cleaned with a 30% isopropyl alcohol solution.

### 2.3 Anxiety behavior: elevated plus maze

The elevated plus maze test was performed to evaluate changes in baseline anxiety levels (Pellow, Chopin, File, & Briley, 1985). The apparatus was composed of four 30 × 5 cm arms positioned 40 cm above the floor and a center platform (5 × 5 cm), with two arms enclosed by acrylic walls. The testing room was illuminated by incandescent lamps (30 lux in the open arms) and the background noise level remained constant at 60 dB. During testing, subjects were recorded for 10 min, during which the Ethovision XT video tracking software (Noldus, Netherlands) scored the frequency of entries and time spent in each of the four arms and center platform. Distance traveled and speed of movement were also assessed. The experimenter was not present during testing. Testing videos were recorded using Pinnacle video capture software (Corel, Canada), then scored offline for head‐dips in open arms and rearing activity by an experimenter blind to group identity. Following testing, mice were returned to an alternate cage with other tested mice and the apparatus was cleaned with a 30% isopropyl alcohol solution and dried thoroughly.

### 2.4 Anxiety levels: light–dark task

The light–dark task was conducted in order to complement the elevated plus maze as a measure of anxiety. The apparatus consisted of a clear acrylic chamber that was modified to allow for a black acrylic insert. The lighting and background noise inside the test chamber were kept constant at 100 lux and 60 dB, respectively. During testing, time spent in the light and dark portions of the chamber were measured for 10 minutes using automated software (Fusion by AccuScan Instruments, Inc., USA). The experimenter was not present during testing. Following testing, mice were returned to an alternate cage with other tested mice and the apparatus was cleaned with a 30% isopropyl alcohol solution and dried thoroughly.

### 2.5 Repetitive behavior: marble burying

The marble burying test was performed to evaluate changes in repetitive behavior. The apparatus consisted of a clean Allentown mouse cage (27 × 16.5 × 12.5) filled with sanichip bedding to a height of approximately 3 cm. Twenty black 15 mm glass marbles were placed throughout the cage in an equidistant 4 × 5 array (Thomas, Burant et al. 2009). During testing, each mouse was individually placed in the testing cage for 30 min and allowed to bury marbles freely. The experimenter was not present in the room for the duration of the testing period. Following testing the mice were returned to their home cage, while the quantity of marbles buried at least 50%, 75%, 100%, and totally buried was tallied by the experimenter.

### 2.6 Repetitive behavior: nose‐poke assay

The nose‐poke assay was conducted as an additional test of repetitive behavior. The apparatus consisted of a board inserted into a clear acrylic area (40 × 40 × 30 cm), with 16 equidistant holes of 1” diameter and approximately 0.75” depth. During testing, mice were placed individually into the apparatus and the number of nose pokes made during a 10‐min period was measured. A nose poke was counted whenever the nose was extended into the hole as far as the eyes. These were counted by a live observer blinded to experimental condition. The arena was cleaned with 30% isopropyl alcohol between subjects. Following testing, mice were returned to an alternate cage with other tested mice and the apparatus was cleaned with a 30% isopropyl alcohol solution and dried thoroughly.

### 2.7 Motor coordination: rotarod

Rotarod performance was measured to assess changes in motor learning. More recently, it has also been proposed that changes in repetitive behavior are also indicative of acquired repetitive behavior (Rothwell Patrick et al., 2014). The apparatus consisted of a rotating rod that accelerated from 5 to 40 RPM over a 5‐minute trial (Series 8 Rotorod; IITC Inc., Woodland Hills, CA, USA). Subjects were tested for two trials per day for 4 days of testing with an intertrial interval of 60 min. During testing, an experimenter blinded to group identity live scored the amount of time each animal was able to stay on the rotating rod before falling off. Following testing, mice were returned to an alternate cage with other tested mice and the apparatus was cleaned with a 30% isopropyl alcohol solution and dried thoroughly.

### 2.8 Social behavior: social chamber

The social chamber test was performed to evaluate changes in social approach behavior. The testing apparatus consisted of a clear acrylic box divided into three chambers, measuring 60 cm × 40.5 cm × 22.5 cm, divided by a 0.25‐cm‐thick acrylic wall. The two outer chambers measured 20.5 cm × 40.5 cm and the middle chamber measured approximately 18.5 cm × 40.5 cm. In the center of each of the dividing walls was a door that was 10 cm × 5 cm. This protocol was previously described in Nadler et al. (2004). Testing was divided into two parts. In part A, the animal was placed in the center chamber and allowed to explore the chamber for 10 minutes. Black wire‐mesh cylinders were placed in the posterior corners of the chamber. A tall plastic bottle was placed on top of the cylinder to prevent the testing mouse from climbing or overturning the cylinder. The animal was then confined to the middle chamber, whereas the researcher placed the intruder mouse (matched for sex, age, and weight) inside one cylinder and a similar sized black block object in the other. The location of the objects was alternated between subjects to prevent a side bias. The barriers to the side chambers were then removed and the subject was allowed to explore for 10 min. The experimenter was not present during the testing window. Videos were analyzed offline for time and frequency in each of the three chambers and investigatory behaviors at the cylinders.

### 2.9 Social behavior: social partition task

The social partition task was used to provide a complementary social behavior test to the results of the social chamber task. This task was used to measure the frequency and duration of interacting with a familiar versus an unfamiliar mouse. The following methods have been previously described (Spencer et al., 2011). The animals were housed for 24 hr in a cage divided into two chambers by a clear partition with 0.6‐cm‐diameter holes. In the other half of the chamber, a sex‐, age‐, and weight‐matched conspecific was placed and animals remained housed together overnight. The following day, the approaches and time spent at the partition by the experimental mice was measured for 5 min in three different conditions. An observer live scored the duration and frequency of sniffing events by inputting the events into a computer software program Ethom (Shih & Mok, 2000). The first condition was with the “familiar” mouse it was housed with overnight, the “unfamiliar” condition was with a novel mouse, and then the “familiar 2” condition was the mouse it had been housed with overnight.

### 2.10 Learning and memory: trace fear conditioning

The trace fear conditioning task was used to evaluate hippocampal‐dependent memory as previously described (Lugo, Smith, & Holley, 2014; Smith, Gallagher, & Stanton, 2007). The testing apparatus consisted of an operant conditioning chamber approximately 26 × 22 × 18 cm high with two clear acrylic and two metal sides. The floor consisted of a metal grid enabling it to deliver a mild shock. This chamber was located inside a second, sound attenuating chamber. Throughout testing on all days, freezing behavior was recorded using the FreezeFrame 3 automated detection software (Coulbourn; Ohio).

On the first day of testing, animals were transported to the holding room and allowed to acclimate for 30 min. Animals were then taken to a separate testing room and placed in the fear conditioning chamber. The first trial consisted of a 12‐min recording period to obtain baseline information. Animals were then returned to the holding room and the apparatus was cleaned with a 30% isopropyl alcohol solution and dried thoroughly. On the second day of testing, animals were placed inside the chamber and allowed to explore freely for 4 min prior to the pairing of the conditioned stimulus (CS) and unconditioned stimulus (US). In this paradigm, the conditioned stimulus consisted of a 20‐s white noise stimulus (70 dB). This was immediately followed by an 18‐s trace period, then a mild foot shock (2‐s, 0.5 mA) as the unconditioned stimulus. Following a 40‐s intertrial interval (ITI) the CS‐US pairing was repeated. This pairing was repeated a total of six times for a total test time of 840 s. Behaviors such as freezing, running, and jumping were recorded by the observer to ensure the foot shock had been delivered. Animals were then returned to the holding room. On the third day of testing, mice were introduced to a novel context wherein the floor, chamber shape, sound, and smell were altered. A novel floor insert was placed on top of the metal grid, the chamber was altered to be a triangle shape by inserting two clear acrylic walls, a fan was turned on to provide background noise, and the smell was altered by placing a small weigh boat of vanilla extract (Adam's extract) under the floor. Animals were exposed to four 100‐s trials, which consisted of an introductory 20‐s interval with no stimuli, followed by a 20‐s presentation of the CS. Each trial was separated by a 60‐s interval with no stimuli prior to the next trial. Animals were then returned to the holding room with other tested mice and the apparatus was cleaned with a 30% ethanol solution and dried thoroughly. On the fourth day of testing, animals were placed in the original context and allowed to explore freely for 3 min as a test of contextual fear conditioning. Experimenters were not present during the testing window.

### 2.11 Learning and memory: delayed fear conditioning

As a complement to trace fear conditioning, we also evaluated a separate cohort of subjects on the delayed fear conditioning task. The delayed fear conditioning paradigm is known to be selective for amygdala‐based fear memories, whereas trace is selective for hippocampal‐based fear memories (Raybuck & Lattal, 2011). The apparatus used in this protocol was the one previously described for trace fear conditioning. Throughout testing on all days, freezing behavior was recorded using the FreezeFrame 3 automated detection software (Coulbourn; Ohio).

On the first day of testing, the animals received two pairings of a 30‐s, 80‐dB white noise (CS) followed immediately by a 2‐s 0.7 mA shock stimulus (US). Following the second pairing, there was a 20 s interval. This trial lasted for a total of 334 s. The second day of testing consisted of two trials. On the first trial, the animal was placed in the familiar context and allowed to move freely for 300 s to evaluate freezing behavior in the original context. After a two‐hour period, the animal was presented with a second trial. For the second trial the context was altered as described above by changing the shape and floor of the chamber as well as a novel vanilla‐scented odor placed under the floor grid. The animal was placed in a new context for 360 s. During the first 3 min the subject was allowed to acclimate to a novel context. During the second 3 min of this trial, the animal was presented with the CS continuously for 3 min and freezing behavior was examined. Experimenters were not present during the testing window.

### 2.12 Statistical analysis

All data were analyzed using GraphPad Software 6.05 (San Diego, CA, USA) or IBM SPSS Statistics 23 (Aramonk, NY, USA). Results were evaluated using a two‐way (Genotype [wild‐type, knockout] × Sex [male, female]) analysis of variance (ANOVA) on each variable for the specific test. Analysis of all results except for the light–dark task involved repeated measures. Information regarding the within‐subjects variable can be found in the specific section. Significant interactions were followed up by formation of a unique grouping variable, “group”, which divided subjects into four groups: male WT, male KO, female WT, and female KO. These interactions were examined with Tukey's LSD post hoc multiple comparisons. For all comparisons, the level of significance remained at *p *<* *.05. Animals were monitored throughout the experiment for weight and no significant differences were found.

## 3 RESULTS

### 3.1 Activity levels: open field

**Figure 1:**
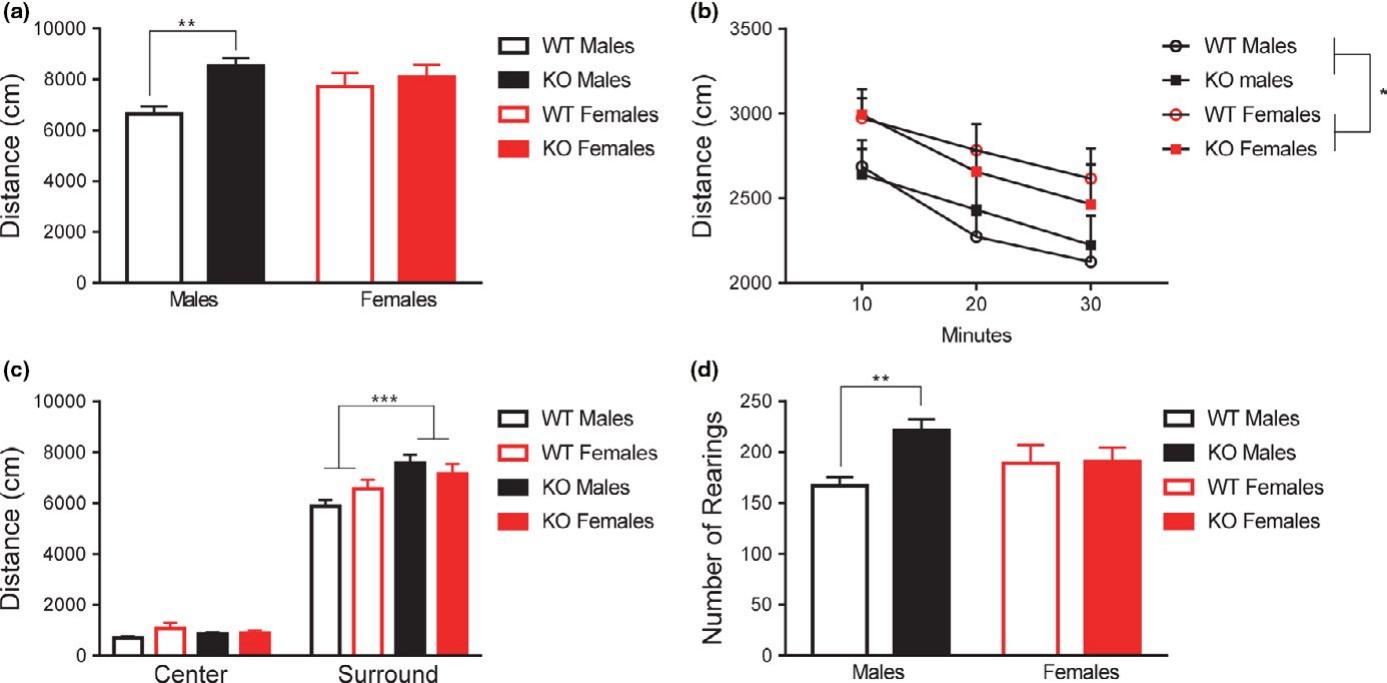
Deletion of *Fmr1* resulted in sex‐specific hyperactivity in the open field task. (a) *Fmr1* KOs showed significant hyperactivity when examining distance moved in the open field task. Upon further statistical analysis, hyperactivity was only detected in the male knockouts. (b) When exploratory behavior was analyzed in 10‐min epochs, *Fmr1* knockouts show similar degrees of habituation during the testing window, however, are more active overall. (c) *Fmr1* KOs show increased activity levels specifically in the periphery of the testing apparatus. (d) Male *Fmr1* KOs exhibited significantly higher amounts of rearing behavior compared to wild‐types, whereas this effect was not present in females. Data are presented as mean ± SEM. ** = *p* < .01, *** = *p* < .001

We observed sex‐dependent differences in activity levels in *Fmr1* knockout mice. The two‐way ANOVA revealed a main effect of genotype*, F*_1,58_ = 7.76, *p *<* *.01, on total distance moved during the 30 min testing window, with *Fmr1* KOs showing increased total distance moved. Neither the main effect of sex, *F*_1,58_ = 0.63, *p* = .43, nor the interaction of sex and genotype, *F*_1,58_ = 3.49, *p* = .07, were significant. However, given the trending interaction, subjects were subdivided into four groups: male WT, male KO, female WT, and female KO, for analysis using *post‐hoc* Tukey's LSD multiple comparisons (Figure 1a). Only males displayed genotype‐specific hyperactivity, *p *<* *.01, whereas females did not, *p* = .53. Taken together, male *Fmr1* knockout mice were hyperactive in the open field testing.

We next wanted to examine if this hyperactivity was a result of failure to habituate over the testing window. A within‐subjects variable of “epoch” was created detailing the total distance moved in ten minute time bins. Results for the repeated measures ANOVA indicated a main effect of epoch, *F*_2,116_ = 23.16, *p *<* *.001. However, this variable did not interact significantly with any between‐subjects factor: genotype, *F*_2,116_ = 0.08, *p* = .93, sex, *F*_2,116_ = 0.05, *p *<* *.95, nor the interaction of sex and genotype, *F*_2,116_ = 0.97, *p* = .38. Taken together, these results indicate all groups showed normal habituation profiles. Between‐subjects results indicated that *Fmr1* KOs exhibit hyperactivity during the entire testing period, *F*_1,58_ = 6.20, *p *<* *.05 (Figure 1b). No main effect of sex was detected, *F*_1,58_ = 0.003, *p* = .96, nor a significant interaction of sex and genotype, *F*_1,58_ = 0.31, *p* = .58.

Next, we examined anxiety behavior in the open field by measuring the location of movement in the center and surround regions of the testing field. We used a within‐subjects variable of “location” to measure distance moved in the center and surround of the field. Results for the repeated measures indicated a significant interaction of genotype and location, *F*_1,58_ = 17.48, *p *<* *.001. Location did not significantly interact with sex, *F*_1,58_ = 0.07, *p* = .80, and the three‐way interaction of location, sex, and genotype was not significant, *F*_1,58_ = 1.88, *p* = .18. Between‐subjects effects indicated a main effect of genotype, *F*_1,58_ = 7.87, *p *<* *.01. No significant effect of sex, *F*_1,58_ = 0.69, *p* = .41, was detected. The interaction of sex and genotype was trending, *F*_1,58_ = 3.43, *p* = .07. To further examine the within‐subjects interaction of location and genotype, the impact of genotype was assessed on each location independently. Results indicated *Fmr1* KOs exhibited higher amounts of locomotion in the surround than WTs, *F*_1,61_ = 13.28, *p *<* *.01, but exhibited similar amounts of movement in the center, *F*_1,61_ = 0.0001, *p* = .99 (Figure 1c).

Similar to overall movement, results of a two‐way ANOVA for rearing behavior indicated that *Fmr1* KO females did not show the same rearing behavior as KO males, compared to WTs. Two‐way ANOVA analysis revealed a main effect of genotype, *F*_1,58_ = 4.90, *p *<* *.05, however, no main effect of sex was noted, *F*_1,58_ = 0.11, *p* = .74. There was a significant interaction of sex and genotype, *F*_1,58_ = 4.31, *p *<* *.05. To further investigate the significant interaction, a unique grouping variable was created to divide subjects into four groups: male WT, male KO, female WT, and female KO. Post hoc LSD analysis on this variable indicated that male KOs showed sex and genotype specific increases in rearing behavior, at the level of *p *<* *.001, whereas female KOs did not, *p* = .93 (Figure 1d).

We also observed differences in time the mice spent performing stereotyped behaviors in the open field, an indicator of repetitive behavior. Two‐way ANOVA for sex and genotype effects testing revealed an overall effect of genotype on stereotyped behavior, *F*_1,58_ = 8.07, *p *<* *.01, with *Fmr1* KOs (25.9 ± 1.5 s) spending more time engaged in stereotypic behavior than WTs (19.9 ± 1.5 s). No main effect of sex, *F*_1,58_ = 0.08, *p* = .77, or interaction of sex and genotype, *F*_1,58_ = 0.13, *p* = .72, was detected.

### 3.2 Anxiety behavior: elevated plus maze

To examine differences in anxiety, as well as exploratory behavior, subjects were also evaluated in the elevated plus maze task. Two‐way ANOVA analysis for main effects of genotype and sex revealed a significant main effect of genotype, *F*_1,58_ = 14.19, *p *<* *.001, where *Fmr1* KOs exhibited higher velocity (5.4 ± 0.7 cm/s) compared to WTs (4.8 ± 0.7 cm/s). No significant main effect of sex was noted, *F*_1,58_ = 0.18, *p* = .67, nor an interaction, *F*_1,58_ = 0.86, *p* = .36.

**Figure 2:**
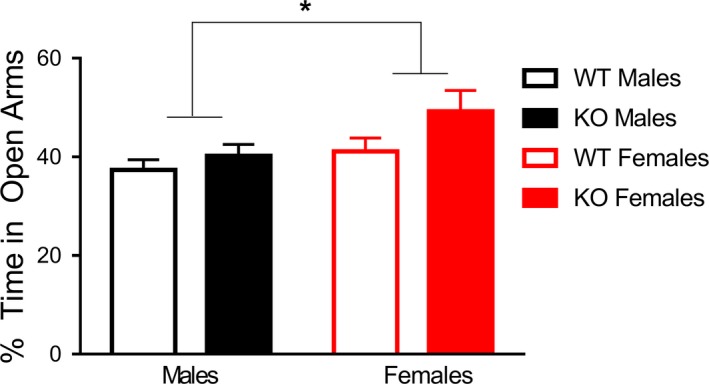
Females displayed decreased anxiety in the elevated plus maze task, independent of genotype. Females across genotypes display less anxiety than their male counterparts, as females spent more time in the open arm as a group compared to males. Data are presented as mean ± SEM. * = *p* < .05

To analyze the proportion of time spent in the various arms of the maze, we then used a two‐way repeated measures ANOVA with a within‐subjects variable noted as “location” including: duration in the open and closed arms. A significant within‐subjects effect of location was noted, *F*_1,58_ = 19.52, *p < *.001. A trending interaction of genotype and location was noted, wherein KOs spent more time in open arms, *F*_1,58_ = 3.29, *p* = .075. Sex and location interacted significantly, indicating that females spent more time in closed arms than did their male counterparts, *F*_1,58_ = 4.27, *p < *.05. There was no significant three‐way interaction of sex, location, and genotype, *F*_1,58_ = 1.08, *p* = .30. Tests of between‐subjects variables indicated a trending effect of genotype *F*_1,58_ = 3.76, *p* = .06 (Figure 2). No effects of sex, *F*_1,58_ = 2.03, *p* = .16 or interaction of sex and genotype, *F*_1,58_ = 0.004, *p* = .95, were noted. No differences were noted in frequency of visits to the various arms, suggesting these effects were not related to hyperactivity (data not shown).

### 3.3 Anxiety behavior: light–dark task

Subjects were evaluated in the light–dark chamber task. Results were evaluated by a two‐way ANOVA on duration spent only in the light portion of the light–dark task. The main effect of genotype was not significant for the light portion, *F*_1,49_ = 0.091, *p* = .76 (WT = 275.6 ± 12.6 s, KO = 270.8 ± 9.5 s). There was no main effect of sex for the duration in the light, *F*_1,49_ = 2.40, *p* = .13 (M = 261.0 ± 10.1 s, F = 285.4 ± 12.1 s). The interaction of genotype and sex was not significant, *F*_1,49_ = 1.13, *p* = .30, for duration spent in the light portion.

### 3.4 Repetitive behavior: marble burying

To examine differences in repetitive behaviors, subjects were tested on the marble burying assay. Results were evaluated using a repeated measures ANOVA with a within‐subjects variable of “percent of burial”. Results of the between‐subjects effects reveal no main effect of genotype *F*_1,58_ = 0.17, *p* = .58, sex *F*_1,58_ = 0.5, *p* = .5, or interaction *F*_1,58_ = 0.4, *p* = .5. Results of the within‐subjects analysis indicated that genotype, sex, and percent buried significantly interacted, *F*_3,174_ = 3.40, *p *<* *.05. There was also a significant two‐way interaction of sex and percent buried, *F*_3,174_ = 2.83, *p < *.05. To further inspect the three‐way interaction, an ANOVA for each of these variables was run with a unique identifier to separate the individual group combinations. No significant differences were detected.

### 3.5 Repetitive behavior: nose‐poke assay

**Figure 3:**
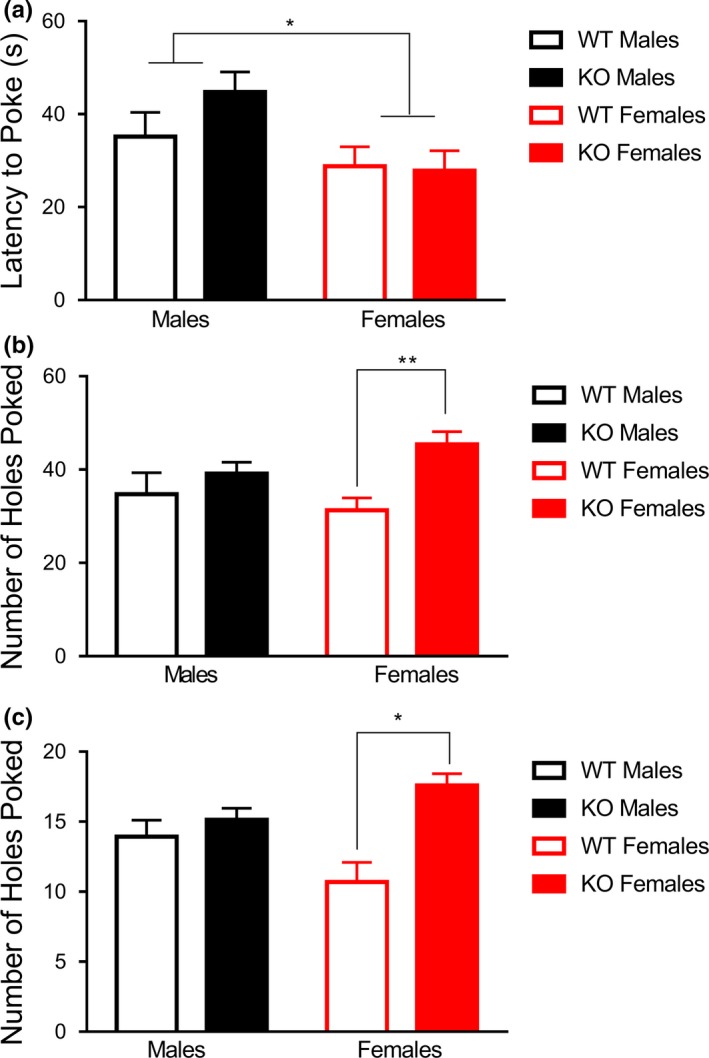
Deletion of *Fmr1* resulted in sex‐specific increases in repetitive behavior across two tasks. (a), Females, independent of genotype, showed a shortened latency to initiate a nose poke than their male counterparts. (b) Analysis of the hole‐poking behavior as a function of center versus surround demonstrated that female *Fmr1* KOs displayed an increase in hole‐poking behavior in the outer holes, whereas male *Fmr1* KOs did not display this increase (c) Female *Fmr1* KOs exhibited higher amounts on hole‐poking behavior on corner holes poked, whereas male *Fmr1* KOs did not differ significantly from male WTs. Data are presented as mean ± SEM.*  = *p* < .05; ** = *p* < .01

The nose‐poke test was used to determine changes in repetitive behavior. Results were first evaluated with a two‐way ANOVA on latency to first hole poke. The results for latency to first nose‐poke detected no main effect of genotype, *F*_1,55_ = 0.91, *p* = .34. There was a significant main effect of sex, *F*_1,55_ = 6.61, *p *<* *.05, with males exhibiting a longer latency to the first hole poke than females (Figure 3a). There was no interaction of genotype and sex, *F*_1,55_ = 1.35, *p* = .25.

We next wanted to analyze if hole‐poking behavior differed between center and surround, a measure of both anxiety behavior and repetitive behavior. A repeated measures ANOVA with a within‐subjects variable of “location”, accounting for the number of hole pokes in the center and the outer holes. A significant three‐way interaction of location, genotype, and sex was noted, *F*_1,52_ = 4.65, *p *<* *.05. Upon further inspection using post hoc LSD, it was noted that female KOs spent significantly more time exhibiting hole‐poking behavior in the surround that did female WT counterparts, *p *<* *.01, whereas males did not show a genotype effect, *p* = .65 (Figure 3b). A significant between subjects effect of genotype was also noted, showing that KOs exhibited more repetitive behaviors overall, *F*_1,52_ = 5.45, *p *<* *.05, which was most likely being driven by the female genotype effect.

Finally, to examine if the distribution of hole‐poking behavior differed on other measures, a repeated measures ANOVA was run with a within‐subjects variable of “location”, made up of front holes poked, back holes poked, and corner holes poked. The three‐way interaction of location, genotype, and sex was not significant, *F*_2,104_ = 1.95, *p* = .15, nor was the interaction of location and genotype, *F*_2,104_ = 1.72, *p* = .18. The interaction of location and sex was also not significant, *F*_2,104_ = 1.90, *p* = .15. A significant between‐subjects interaction of sex and genotype was noted, with female KOs exhibiting more hole‐poking behavior than any other group on each of these measures, *F*_1,52_ = 6.81, *p *<* *.05 (Figure 3c). A main effect of genotype, *F*_1,52_ = 8.37, *p *<* *.01, was also noted, whereas the main effect of sex was not significant, *F*_1,52_ = 0.03, *p* = .86.

### 3.6 Motor coordination: rotarod

To examine changes in motor learning, coordination, and repetitive behavior, subjects were tested in the accelerating rotarod task. A two‐way ANOVA with repeated measures for latency to fall on each of the eight trials revealed a trending interaction of genotype and trial on latency to fall across the eight trials, *F*_1,45_ = 1.98, *p* = .06. This was further demonstrated by a significant linear interaction contrast of trial and genotype, *F*_1,45_ = 4.90, *p *<* *.05. Between subjects effects demonstrated a significant main effect of sex, *F*_1,45_ = 7.41, *p *<* *.01, with females exhibiting a higher latency to fall.

**Figure 4:**
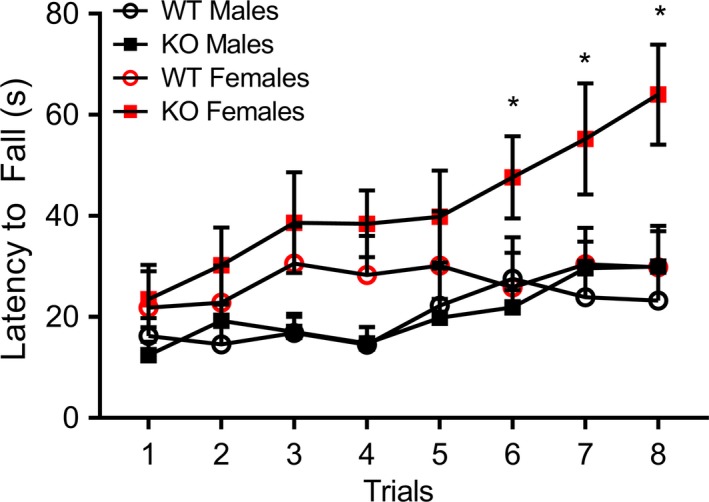
*Fmr1* knockout females display enhanced motor coordination on the accelerating rotarod task. Female KO mice showed enhanced latency to fall on later trials of the accelerating rotarod task. Data are presented as mean ± SEM. * = *p* < .05

Given these interactions, we next created a unique identifying variable called “group”, such that each combination (male WT, male KO, female WT, and female KO) was analyzed independently. A second repeated measures analysis using this variable indicated a significant interaction linear contrast of trial and group, *F*_3,45_ = 3.03, *p *<* *.04. A significant main effect of group was also noted, *F*_3,45_ = 4.67, *p *<* *.006. To further investigate these effects, post hoc LSD multiple comparisons were performed separately for each trial, comparing across the four groups. Post hoc LSD tests indicated that beginning on trial 6, female KOs performed significantly better than all three other comparison groups, at the level of *p *<* *.05 (Figure 4).

### 3.7 Social behavior: social chamber

Following testing in the previous task, one cohort of subjects was tested in the three‐chambered social apparatus. Using offline scoring blind to group, duration in each of the three chambers for phase A and B, as well as time at both cups was recorded. In order to test for a side bias, results for Phase A for the left and right chamber were tested for differences in duration of test time spent in the respective chambers, using a repeated measures analysis with a within‐subjects variable of “chamber”. The three‐way interaction of chamber, sex, and genotype was not significant, *F*_1,56_ = 0.05, *p* = .82, nor was the interaction of sex and chamber, *F*_1,56_ = 1.96, *p* = .17. The analysis indicated a significant within‐subjects interaction of chamber and genotype, *F*_1,56_ = 6.39, *p *<* *.05. Further investigation using individual ANOVAs for each chamber indicated that KOs spent less time proportionally in the right chamber, *F*_1,59_ = 8.50, *p *<* *.01, compared to the left, *p *>* *.05. No significant between‐subjects effects were detected: genotype, *F*_1,56_ = 2.20, *p* = .14, sex, *F*_1,56_ = 0.06, *p* = .81, genotype x sex, *F*_1,56_ = 0.06, *p* = .81.

To correct for the detected side bias, the location of the conspecific‐containing cup was altered on each successive trial. Results for Phase B were analyzed by a repeated measures ANOVA, with the within‐subjects variable of chamber accounting for duration of time spent in the chamber housing the conspecific and the novel object cup. Results indicated no within‐subjects effects, and the three‐way interaction of sex, genotype, and chamber was not significant, *F*_1,56_ = 0.15, *p* = .70. Rather, KOs spent more time investigating overall, indicated by the main effect of genotype, *F*_1,56_ = 6.53, *p *<* *.05. No main effect of sex, *F*_1,56_ = 2.19, *p* = .15, or interaction of sex, *F*_1,56_ = 0.33, *p* = .57, was indicated.

Results for the duration of time spent investigating the cups containing the novel conspecific and the object were analyzed using a repeated measures ANOVA, with a within‐subjects variable of location. Results indicated no significant within‐subjects interactions, and the three‐way interaction of sex, genotype, and location was not significant, *F*_1,56_ = 0.05, *p* = .83. Results for between subjects factors indicated no significant effects of genotype, *F*_1,56_ = 0.02, *p* = .90, or sex, *F*_1,56_ = 0.36, *p* = .55. The interaction of sex and genotype was not significant, *F*_1,56_ = 0.004, *p* = .95.

### 3.8 Social behavior: social partition task

As a complement to the three‐chambered social task, another cohort of animals was tested in the social partition paradigm. Results for the three trials were evaluated with a repeated measures ANOVA with a within subjects variable of “trial”, consisting of the duration of time at the partition for each of the three trials. Tests for within subjects effects revealed no significant effects, and the three‐way interaction of sex, genotype, and trial was not significant, *F*_2,90_ = 2.09, *p* = .13. Results for between subjects effects revealed no significant impact of genotype, *F*_1,45_ = 1.14, *p* = .24 (WT = 67.0 ± 10.3 s, KO = 82.6 ± 7.9 s) across the three trials. There was also no effect of sex, *F*_1,45_ = 0.001, *p* = .98 (M = 74.6 ± 8.2 s, F = 75.0 ± 10.0 s) on duration of time spent at the partition. The interaction of sex and genotype was also not significant, *F*_1,45_ = 0.29, *p* = .59 across the three trials.

The same pattern was noted for frequency of visits across the three trials, analyzed the same way. No within‐subjects effects were noted, and the three‐way interaction of trial, sex, and genotype was not significant, *F*_2,90_ = 0.16, *p* = .86. The between subjects effect of genotype, *F*_1,45_ = 2.09, *p* = .16 (WT = 11.3 ± 1.0 s, KO = 13.2 ± 0.8 s) was not significant. Nor was the effect of sex, *F*_1,45_ = 1.05, *p* = .31 (M = 11.6 ± 0.8 s, F = 12.9 ± 1.0 s).

### 3.9 Learning and memory: trace fear conditioning

Following testing for social partition, subjects were evaluated in trace fear conditioning as a test of hippocampal‐based fear memory. On Day 1, subjects revealed no effect of genotype, *F*_1,58_ = 2.0, *p* = .11, sex, *F*_1,58_ = 0.15, *p* = .9, or interaction *F*_1,58_ = 0.45, *p* = .83 across the 12‐min testing trial. On Day 2, results were analyzed using a two‐way ANOVA with repeated measures for the six instances of the trace period. A significant main effect of genotype was detected, *F*_1,58_ = 6.49, *p *<* *.05, with *Fmr1* KOs freezing significantly less than WTs across time (Figure 4a). There was a no main effect of sex, *F*_1,58_ = 0.000, *p* = .99. No significant interaction of genotype and sex was detected, *F*_1,58_ = 0.24, *p* = .63.

**Figure 5:**
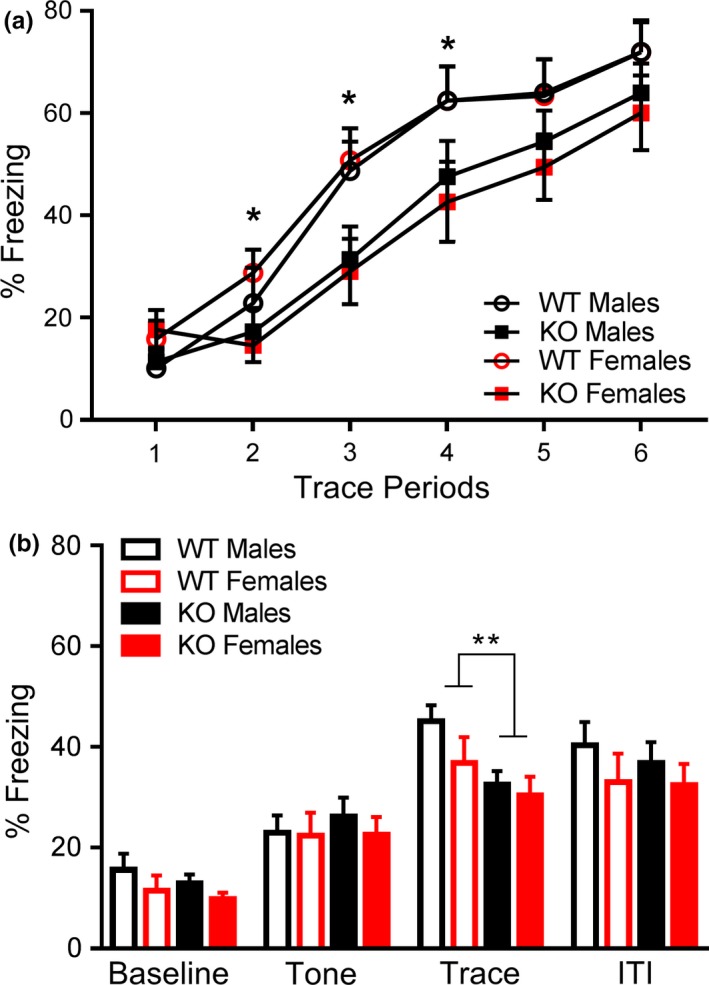
*Fmr1* knockouts displayed decreased learning in the trace fear conditioning task. (a) *Fmr1* KOs exhibited less freezing behavior during the acquisition of the fear response across the six trace periods. (b) *Fmr1* KOs exhibited less freezing during the trace period in a novel testing environment. Data are presented as mean ± SEM. * = *p* < .05; ** = *p* < .01

On Day 3, cued fear conditioning was tested in a novel environment. During this task, the tone‐trace period‐ITI bout was repeated four times. We used the mean for each time point and analyzed the results with a two‐way ANOVA with repeated measures. There was no effect of genotype, *F*_1,58_ = 0.85, *p* = .36, sex, *F*_1,58_ = 1.6, *p* = .21, or genotype x sex interaction, *F*_1,58_ = 0.7, *p* = .78. There was a significant difference in freezing over the four instances of the trace period, *F*_3,174_ = 122.8, *p *<* *.001 and there was a significant interaction between group over the 4 period *F*_3,174_ = 5.3, *p *<* *.01. Separate individual t‐tests revealed reduced freezing in the KO mice in the trace period, *t*_60_  = 2.7, *p *<* *.01 compared to the WT mice (Figure 5b). No other statistical differences between the groups were noted.

On Day 4, subjects were returned to the training environment (context conditioning) to evaluate hippocampal memory. Results were analyzed using the two‐way ANOVA with repeated measures. The three‐way interaction of sex, genotype, and time was not significant, *F*_2,116_ = 0.189, *p* = .83. No interaction of genotype and time was detected, *F*_2,116_ = 0.27, *p* = .76. There was a significant interaction of sex and time, *F*_2,_116 = 4.32, *p *<* *.05. No significant interaction between subjects effects were detected: main effect of genotype, *F*_1,58_ = 0.3, *p* = .64; main effect of sex, *F*_1,58_ = 0.03, *p *=* *.87. No significant interaction was detected, *F*_1,58_ = 0.22, *p* = .64. To follow‐up on the interaction of sex and time, independent ANOVAs were run for each minute tested. Results indicated that females exhibited significantly less freezing behavior in the first minute, *F*_1,61_ = 5.52, *p *<* *.05.

### 3.10 Learning and memory: delayed fear conditioning

**Figure 6:**
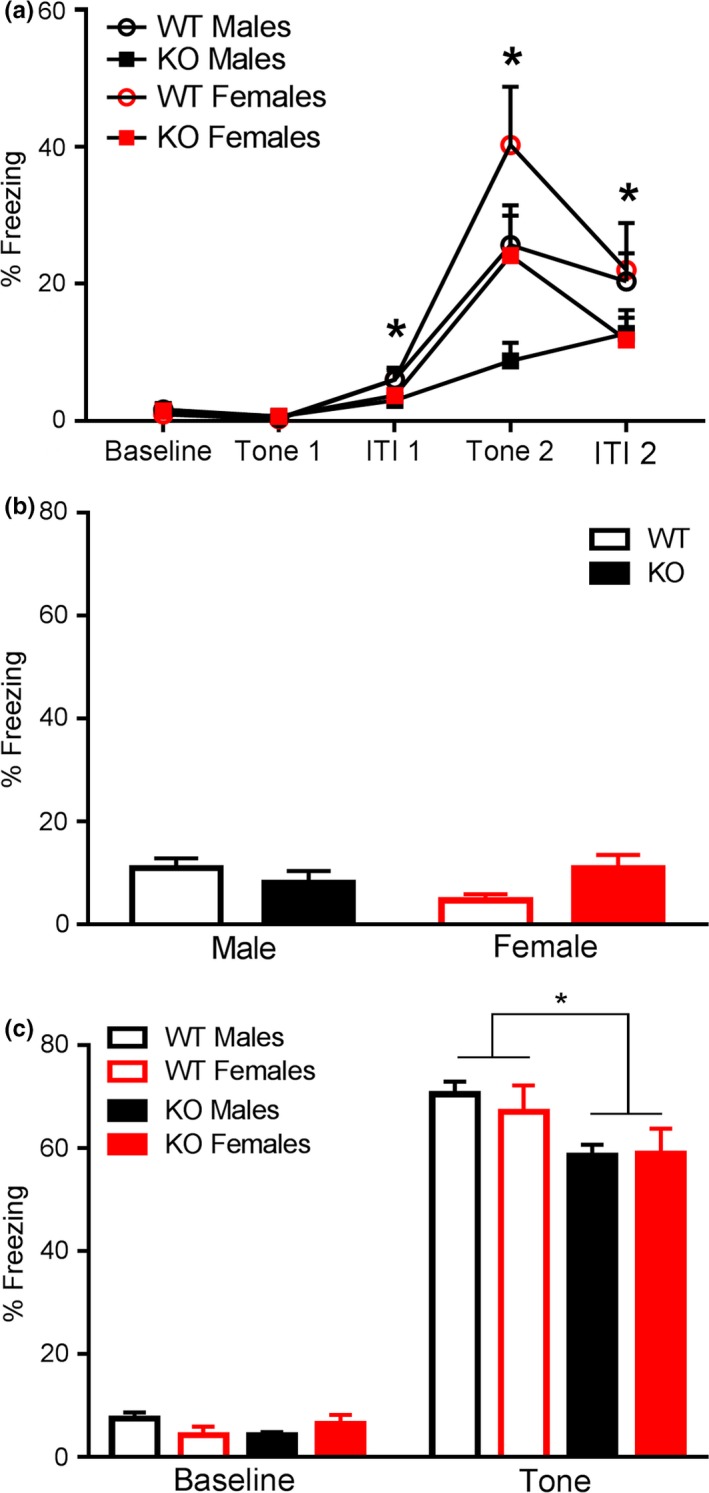
*Fmr1* knockouts displayed decreased learning and memory capabilities in the delayed fear conditioning task. (a) *Fmr1* KOs presented significantly decreased freezing in response to the first intertrial interval (ITI), second CS presentation and second ITI. (b) No significant differences by sex and genotype were detected in contextual fear conditioning. (c) *Fmr1* KOs exhibited significantly reduced freezing to presentation of the CS in a novel context. Data are presented as mean ± SEM. * = *p *< .05

A separate cohort of animals was examined in the delayed fear conditioning task, as a complement to the trace fear conditioning trials. On Day 1, subjects were presented with repeated pairings of the CS and US stimuli. Results were analyzed using a two‐way ANOVA with repeated measures. The within‐subjects variable was defined as “time” with five levels: baseline, tone 1, intertrial interval 1, tone 2 and intertrial interval 2. The three‐way interaction for sex, genotype, and time was not significant, *F*_4,180_ = 0.06, *p* = .99. Results for within‐subjects effects did indicate a significant within‐subjects interaction of time and genotype over the five testing periods, *F*_4,180_ = 5.98, *p *<* *.001. To further analyze the significant interaction of genotype and time, results were analyzed using an ANOVA for genotype on each time point. Results indicated KOs froze less during the first ITI, *F*_1,48_ = 6.24, *p *<* *.05, during the second presentation of the tone, *F*_1,48_ = 6.86, *p *<* *.05, and during the second ITI, *F*_1,48_ = 4.59, *p *<* *.05 (Figure 6a). There were no differences in freezing at baseline or during the 1st presentation of the CS. A significant between‐subjects effect of genotype was also detected, *F*_1,45_ = 9.73, *p *<* *.01, with *Fmr1* KOs freezing less over time. There was no between‐subjects effect of sex, *F*_1,45_ = 2.96, *p* = .09. There was also no significant between‐subjects interaction of sex and genotype, *F*_1,45_ = 0.0004, *p* = .98. Taken together, these results indicate that *Fmr1* KOs, independent of sex, failed to acquire freezing behavior in response to the tone/shock pairing. There was also a significant within‐subjects interaction of sex and time, F_4, 180_ = 5.16, *p *<* *.05. Follow‐up analysis indicated that females, independent of genotype, showed enhanced freezing behavior during the second presentation of the tone, *F*_1,48_ = 5.54, *p *<* *.05.

On Day 2 of testing, animals were placed in a familiar context and freezing behavior was evaluated in the 5‐min trial. We used a Kruskal–Wallis test to analyze the groups because there was not homogeneity of variance across the groups. Using a two‐way ANOVA revealed no significant effect of genotype, *F*_1,45_ = 0.47, *p *=* *.50, or sex, *F*_1,45_ = 0.48, *p* = .49. There was a trending interaction of sex and genotype, *F*_1,45_ = 3.23, *p* = .08 (Figure 6b). However, further multiple comparisons yielded no discernible pattern.

In the second part of testing for Day 2, animals were placed in an unfamiliar context and freezing behavior to the CS as well as at baseline was evaluated. Results indicated that *Fmr1* KOs performed poorly on tests of cued fear conditioning. A repeated measures ANOVA was conducted. The within‐subjects variable of time had two levels: baseline percent freezing and percent freezing during the tone presentation. No three‐way interaction of time, genotype, and sex was detected, *F*_1,45_ = 0.05, *p* = .83, nor was an interaction of sex and time, *F*_1,45_ = 0.09, *p* = .76. Results for within‐subjects effects indicated that there was as significant interaction between genotype and time *F*_1,45_ = 7.9, *p *<* *.01 (Figure 6c), suggesting that *Fmr1* KOs, independent of sex, behaved differently over time. To further investigate these results, independent ANOVAs were run to analyze the impact of genotype at baseline and during tone presentation separately. Results revealed during the presentation of the tone, *Fmr1* KOs spent significantly less time freezing than WTs, *F*_1,45_ = 6.83, *p *<* *.05. This effect was not due to hyperactivity, as no significant main effect of genotype was detected at baseline, *F*_1,45_ = 0.15, *P* = .70. Results for between subjects effects revealed a significant main effect of genotype, *F*_1,45_ = 5.05, *p *<* *.05. The main effect of sex was not significant, *F*_1,45_ = 0.20, *p* = .66, nor was the interaction of genotype and sex, *F*_1,45_ = 0.92, *p* = .34.

## 4 DISCUSSION

The purpose of this study was to characterize the effect of *Fmr1* deletion in male and female animals. Previous studies evaluating the impact of *Fmr1* deletion often do not include female animals, whether due to avoidance of potentially confounding variables or to the high cost of potentially doubling sample sizes. Our data show that the deletion of *Fmr1* produces sex‐specific behavioral changes. Female *Fmr1* KOs showed increased repetitive behaviors on both the nose‐poke task and enhanced motor coordination on the accelerating rotarod when compared to their female WT counterparts, whereas males KOs lacked a similar effect. Social behavior in both the three‐chambered social task and social partition task was unaffected across sexes. Deletion of *Fmr1* also resulted in learning and memory deficits in both trace and delayed fear conditioning paradigms across both sexes. Hyperactivity was detected in the open field task, but only in male KO mice.

Previous examinations of repetitive behavior phenotypes in *Fmr1* KO mice have yielded few statistically significant results. Moreover, most of these effects have been weak and nonreproducible across strains. In a seminal article where the investigators generated six *Fmr1* KO mouse strains, increases in marble burying were observed in only one of the tested strains, though no effect of *Fmr1* deletion on marble burying behavior was noted across all strains. One other paper found a deficit in marble burying behavior that was only trending toward a statistically significant difference (Veeraragavan et al., 2011). In both studies only males were used, whereas our study reports the novel finding of female‐specific increases in the repetitive behavior of *Fmr1* KOs on the nose‐poke task. Therefore, the conclusion that *Fmr1* KO mice do not have alterations in repetitive behavior may only be representative of male KOs. In support of this assertion, authors of one previous study briefly mentioned similar female‐specific increases in nose‐poke behavior, though these results were not highlighted (Baker et al., 2010).

Female *Fmr1* KOs also showed increased latency to fall in the accelerating rotarod task. The rotarod task is often regarded as a test of cerebellar coordination and motor ability. Previous research has reported no differences in the latency to falling in the *Fmr1* KO compared to WT (Heulens, D'Hulst, Van Dam, De Deyn, & Kooy, 2012; Peier et al., 2000; Spencer et al., 2011). However, one previous investigation has indicated that female wild‐type mice show improved performance on the accelerating rotarod task across multiple strains (McFadyen, Kusek, Bolivar, & Flaherty, 2003). Phenotypic analyses of another monogenic model of ASD, the neuroligin‐3 (*NLGN‐3*) knockout mouse, have noted enhanced motor learning in the rotarod task, similar to our *Fmr1* KO females (Rothwell Patrick et al., 2014). The authors from Rothwell Patrick et al., 2014 suggested that several components of the motor routine become less variable with training such that latency to falling in this task could be considered an indicator of acquired repetitive behavior. In accordance with these results, we suggest that perhaps female *Fmr1* KOs display increases in acquired repetitive behavior in this task.

Hyperactivity in animal models is often considered a confound behavior that could be driving performance in the rotarod task, however, in this study, this is not the case for two reasons. Given the robust findings in the open field task, one would expect that there would be a significant difference on the first trial, which we did not observe. Secondly, female *Fmr1* KO mice had similar levels of activity compared to WT mice in the open field test, but showed enhanced accelerating rotarod performance. This evidence is congruous with clinical data in humans suggesting that FXS‐related hyperactivity in females is less common than in FXS males (Freund, Reiss, & Abrams, 1993).

Previous investigations in *Fmr1* male KOs often show decreased sociability in both social partition and the three‐chambered social task (Liu & Smith, 2009; Moy et al., 2009; Pietropaolo, Guilleminot, Martin, D'Amato, & Crusio, 2011), which is consistent with ASD and FXS symptomologies. As such, a surprising finding of this study was that *Fmr1* KOs show no change in investigation time in social tasks across both sexes. One other study reported similar social preference in the three‐chambered social task between *Fmr1* WT and KO mice (McNaughton et al., 2008). These discrepancies may be as a result of a variety of environmental and methodological factors. For example, when behavior in the social partition is examined over time bins, *Fmr1* KO mice show initial suppression of social investigation during the first 5 minutes of the task, followed by enhanced investigation in the later part of the task (Spencer, Alekseyenko, Serysheva, Yuva‐Paylor, & Paylor, 2005). Assays of social behavior in this model may be influenced by cage familiarity. On the first day of testing, in an unfamiliar cage, *Fmr1* KOs exhibited similar time spent at the partition. During the second day *Fmr1* KO were presented with new unfamiliar partners in the same (“familiar”) cage. During the second “familiar cage” trial, KOs reacted differently than WTs, and the direction of the effect changed across the four time bins. The authors of this study suggested that the social response of the *Fmr1* KO mice are dependent on experience. Therefore, it is possible that the lack of alterations seen in this study may reflect an adaptation to the cage environment. Future studies could examine other paradigms known to detect such subtleties.

One possible limitation of this study is the lack of assessment of estrous cycle of the females mice used in the study. Given that most of these behavioral paradigms only span one day, it is possible that differences could reflect differences in estrous cycles between groups. A recent study comparing heterozygous *Fmr1* mutants did assess the estrous cycle of subjects, however, it was shown that estrous cycle did not significantly impact the results shown (Gauducheau et al., 2017). Furthermore, a recent meta‐analysis of 293 articles found that variability was not greater in females for behavioral tasks when they did not account for the estrous cycle (Prendergast et al., 2014). However, given that these subjects were separately housed, it is possible that these effects could account for changes seen. Future studies should assess this possibility.

Another possible limitation of our study is the absence of *Fmr1* heterozygous female mice, for two reasons. The first is that in human cases of FXS, heterozygous mutations in *FMR1* are most common, with homozygous mutations rarely occurring. Heterozygous females with the full *FMR1* mutation display mosaic expression of FMRP from the unaffected X chromosome. This typically leads to behavioral variability, which can be typically attributed to a gene dosage effect. Previous studies have examined behavior in the heterozygous *Fmr1* female, but few differences were noted between homozygous and heterozygous females, mostly limited to juvenile aged animals (Gauducheau et al., 2017; Qin et al., 2005). Although previous studies have suggested that potential differences in female *Fmr1* KOs, as well as human clinical data, are linked directly to a gene dosage effect, the behavioral differences noted in this study are independent of *Fmr1* expression. Second, the breeding paradigms used to produce wild‐type and homozygous *Fmr1* knockout mice require different pairings, meaning that litter effects could significantly influence behavior (Zupan & Toth, 2008). These effects could be controlled by inclusion of a female heterozygous group and future studies should directly compare these groups. This study does serve to highlight the role of sex as a biological variable mediating the behavioral phenotype following complete loss of *Fmr1* gene expression, independent of gene dosage effects.

The larger scientific community has begun to place a larger emphasis in investigating and understanding the mediational role of sex in the *Fmr1* KO, and more broadly, in biomedical research. Understanding sex as a biological variable has wide‐ranging implications, including enhancing the reproducibility of research (Collins & Tabak, 2014) and the betterment of women's health. Broadly, these findings of this study underscore the importance of including females in preclinical examinations, for the development of potential therapeutic interventions for example. While some have suggested that the female *Fmr1* KOs should be included in studies with males based on similarity, here we highlight the marked phenotypic differences, and as such, future research could focus on how these phenotypes could, and should, be treated separately. For instance, repetitive behavior phenotypes are commonly treated using several different types of drugs, including 5‐HT1BR agonists (Ho et al., 2016), 5HT1A partial antagonists (Chugani et al., 2016) and more recently, antioxidants (Hardan et al., 2012). Through the routine exclusion of females from biomedical studies, opportunities are missed to explore potential treatments and how sex may impact the efficacy of such treatments.

## CONFLICT OF INTEREST

The authors have no conflicts of interest to declare.
